# Growing up with tic disorders: an Italian survey on quality of life and access to care

**DOI:** 10.3389/fpsyt.2025.1581666

**Published:** 2025-05-13

**Authors:** V. Baglioni, D. Esposito, I. Notaristefano, G. Di Iorio, S. Romano, F. Pisani

**Affiliations:** Department of Human Neuroscience, Unit of Child and Adolescent Neuropsychiatry, Sapienza University of Rome, Rome, Italy

**Keywords:** Tourette syndrome, tics, healthcare, quality of life, stigma, access to care

## Abstract

**Background:**

Tourette Syndrome (TS) and chronic tic disorders (TD) are complex neuropsychiatric conditions often associated with comorbidities. Despite their prevalence, these disorders are frequently underdiagnosed and poorly managed due to limited healthcare access and lack of specialized services. An online survey was conducted in Italy to assess access to care and the impact of TS/TD on social, educational, and occupational life.

**Method:**

A nationwide online survey, including 100 participants (mean age 23.1, SD 14.6; M:F=77:33), was developed to assess diagnostic-therapeutic processes and quality of life (QoL) in TS/TD. The survey had three sections: 1) Access to Care, 2) Tic Severity, and 3) Impact of TS/TD.

**Results:**

Diagnosis was delayed by an average of 7.7 years. The lack of clear information was a major issue, with caregivers reporting easier access to information than youths and adults with TS/TD (p= .042, OR: 0.37). Pharmacological treatment was the most common (53% currently, 63% previously), while psychotherapy was more common among children and early adolescents (50%) compared to older participants (25.6%) (p= .037). Specialized cognitive-behavioral treatments, such as EPT and HRT, were rare, with only 7% receiving HRT and none undergoing EPT. Comorbidities had a significantly greater impact on QoL than tics (p= .004, Cohen’s d=0.3).

**Conclusion:**

These findings highlight the need for improved access to specialized care, greater healthcare professional awareness, and enhanced support for individuals with TS/TD and their families, especially for early diagnosis and effective cognitive behavioral treatments.

## 1 Introduction

According to DSM V-TR criteria, Chronic Tic Disorders (CTDs) are neurodevelopmental conditions characterized by the presence of motor and/or vocal tics that persist for at least one year. These tics are sudden, repetitive, and non-rhythmic movements or vocalizations that fluctuate in severity over time. CTDs include Chronic Motor Tic Disorder (CMTD) and Chronic Vocal Tic Disorder (CVTD), distinguished by the presence of either motor or vocal tics, respectively, but not both (1).

Tourette Syndrome (TS) is a more complex tic disorder defined by the presence of both motor and vocal tics persisting for at least one year. TS is often associated with psychiatric comorbidities, including obsessive-compulsive disorder (OCD), attention-deficit/hyperactivity disorder (ADHD), and anxiety, which significantly impact quality of life (QoL) (2). The estimated overall prevalence of TS is 0.23% among all children and adolescents aged 0–17 years (3), and up to 4% for CTDs (4), with males being affected approximately three to four times more frequently than females (5).

The expression of TS can vary significantly among individuals and is frequently accompanied by high rates of comorbid conditions, observed in up to 90% of patients (6, 7). Notably, Attention Deficit Hyperactivity Disorder (ADHD) and Obsessive Compulsive Disorder (OCD) are the most common comorbidities associated with TD (6), however many patients also experience mood disorders, impulse control difficulties, self-injury, or socially inappropriate behaviors (8).

The intensity of tics often peaks in early adolescence, with many individuals experiencing a decline in both frequency and severity after puberty (8). However, approximately 20% of children with TD continue to exhibit moderate functional impairment as they transition into adulthood (9). Despite this, in many countries, a significant concern is the lack of continuity in specialized care during this transition, potentially impacting long-term outcomes (6).

TS/TD have been associated with an impairment in quality of life (QoL), leading to adverse psychological, behavioral, social, and academic outcomes (6, 10, 11). Increased severity of tics, accompanied by the presence of comorbid conditions, significantly contributes to these negative psychosocial effects (12). It is known that sleep disturbances, such as bedtime resistance and fragmented sleep, contribute to heightened physiological and psychological stress in individuals with TS/TD, exacerbating tic severity through increased stress and impaired emotional regulation (13). Also, recent studies suggest that elevated perceived stress and impaired family functioning are among the strongest predictors of reduced QoL in adolescents with TS/TD. Family dynamics, characterized by communication difficulties and decreased cohesion, along with heightened stress sensitivity, may exacerbate emotional and social distress, regardless of tic severity (14). Furthermore, experiences of stigmatization and social rejection can exacerbate challenges, limiting opportunities for developing friendships and essential social skills (6).

Another factor contributing to the reduced QoL in this population is the lack of adequate information regarding the diagnostic and therapeutic pathways (15, 16). This informational gap not only creates barriers to obtaining a timely diagnosis but also limits access to first-line treatments for tics, such as cognitive-behavioral therapy (15).

Studies in Canada (17), the United States (18), Spain (19), and Australia (20) echo similar challenges, noting that the diagnostic process for TS/TD is often lengthy and complex. Additionally, healthcare professionals lack knowledge about tics, and there is a shortage of qualified teams to manage TS/TD and its comorbidities (15, 20).

In light of these discussed concerns, a comprehensive survey across Italy was conducted to investigate access to care, and the accessibility of diagnostic and therapeutic processes in both the National Health Service (NHS) and private facilities. This survey will also assess the long-life impact of TS/TD on social interactions, educational and employment experiences, romantic relationships, and the presence of comorbid conditions.

## 2 Materials and methods

A comprehensive questionnaire was developed to assess various aspects of the therapeutic-diagnostic process for TS/TD. The survey was designed by experienced neurodevelopmental neuropsychiatrists (VB, SR, GDI) and subsequently revised in collaboration with representatives from family associations of individuals with TS/TD (Associazione Tourette Roma Onlus) to ensure a thorough exploration of relevant issues. The final instrument comprised two versions: a 40-item self-report questionnaire for adult participants and a 33-item caregiver-report questionnaire for patients under 18 years of age. In particular, adult respondents completed an assessment of the quality of the transition from pediatric to adult services.

The survey was administered anonymously via Google Forms and yielded 100 responses from across Italy between October and December 2024. The questionnaire was organized into three primary sections:

- Access to Care: Adapted from the European Patients’ Forum (EPF) report (15). This section evaluates challenges related to accessing care for individuals with TS/TD (15).
- Tic Severity Rating Scales: This section is an adaptation of the Yale Global Tic Severity Scale (YGTSS) (21).
- Impact of TS/TD: A self-developed section examining the influence on various facets of life, such as social relationships, educational and occupational challenges, romantic relationships (adults only), social stigma, diagnosed comorbidities, and details regarding current and past therapies.

Participants were recruited nationally through patient association websites, complemented by direct outreach to clinicians who agreed to participate. However, since this is a self-report survey and not all patients were directly recruited by clinicians, we have no way of verifying how the diagnosis of TS/TD was made for the entire sample (e.g., clinician-confirmed diagnosis of tic disorder by DSM-IV-TR criteria). The survey, available in English in the **Supplementary Material** (**Appendix S2**), was administered voluntarily without any compensation.

Baseline demographics and characteristics were summarized descriptively across groups using mean and standard deviation (SD) for continuous variables. Categorical variables were presented as frequencies and percentages. The analysis included inferential bivariate methods (t-test, chi-squared, Fisher’s test). All participants with complete data were included in the analyses (n = 100). For all comparisons, p-values less than 0.05 were considered statistically significant. Statistical analyses were performed using the Jamovi statistical software (version 2.3.28) based on the R language (22, 23).

## 3 Results

### 3.1 Description of the sample

The total sample consisted of 100 individuals, divided into patients (n 33) and caregivers (n 67) (**Table 1**). Participants were stratified into two age groups based on the WHO definition of youth and adolescence (19) using a 15-year threshold: children and adolescents (n 26) and youth and adults (n 74). This categorization was then applied in the statistical analyses to examine age-related differences. The demographic data are presented in **Table 1**.

**Table 1:** Description of the sample divided into youth and adults and children and early adolescents.

Information	Total Sample (n = 100)	Youth and Adults*¹* (n = 74)	Children and Early Adolescence*²* (n = 26)
Caregiver-reports - *%*	67	52.7	100
Current age (years) - *mean (SD)*	23.1 (14.6)	26.5 (15.5)	13.2 (1.6)
Age of TS diagnosis (years) - *mean (SD)*	14.4 (10.4)	16.1 (11.6)*	9.8 (2.9)*
Symptoms onset-TS¹ diagnosis (years) - *mean (SD)*	7.7 (9.7)	9.3 (10.8)*	3.5 (2.9)*
Gender (Male) - *%*	77	71	61.5
Gender identity (cisgender) - *%*	98	100	92.3
Urban area - *%*	70	67.5	80.7

¹Patients older than 14 years of age; ² Patients younger than 14 years of age.

* Statistically significant differences after independent t-tests or chi-squared tests between the two subgroups.

The mean complexity score for tics was 1.42 (SD = 1.04; range 1–3, where 1 represents only simple tics and 3 represents severe tics) for motor tics and 1.17 (SD = 1.05; range 1–3) for vocal tics. Similarly, the mean disability score was 1.43 (SD = 1.20; range 0–4, where 0 represents no disability and 4 represents severe disability) for motor tics and 1.18 (SD = 1.03; range 0–4) for vocal tics. These scores were assessed using an adaptation of the YGTSS.

Independent t-tests indicated that youth and adults were diagnosed with TS/TD significantly later than children and adolescents (p = .012), and also experienced a longer delay between symptom onset and diagnosis (p = .011).

**Figure 1:**
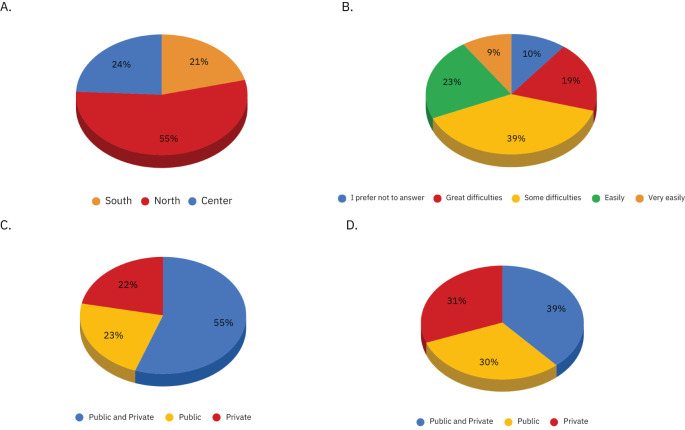
Figure shows the distributions of the following variables: **(A)** Geographical provenance of the sample, **(B)**. Family budget, **(C)**. Method of performing the diagnostic pathway: through the National Health System (NHS) or private facilities, **(D)**. Method of performing the treatment course: through the National Health System (NHS) or private facilities.

Most of the sample reported having some (39%) or great (19%) difficulties in covering medical expenses. (**Figure 1B**).

**Figure 2:**
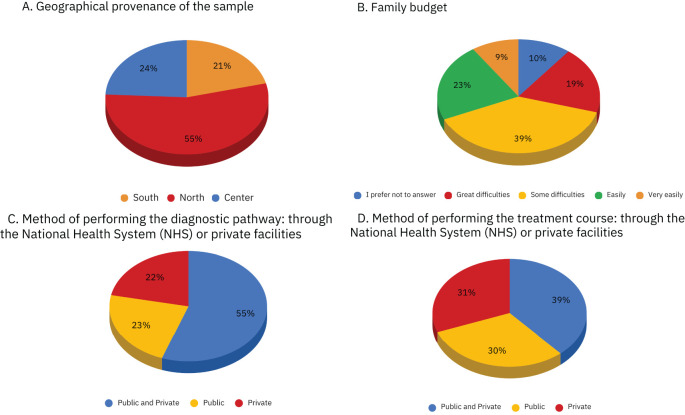
Graphs of the geographical proveneance, family budget, method of performing diagnostic and therapeutic pathway in our sample.

Both established neuropsychiatric diagnoses and comorbid psychopathological symptoms were investigated. The most represented comorbid diagnoses within the sample were ADHD (49%), followed by mood disorder (45%). (**Figure 2A**).

**Supplementary Figure S1** (in the **Supplementary Materials**) shows the frequency of comorbid symptoms within the sample. The most represented were anxiety and depression (35%), followed by inattention (35%), and impulsivity (30%). Only 5% of the respondents reported no other symptomatology besides TS/TD.

### 3.2 Diagnostic/therapeutic process and access to care

More than half of the sample (55%) used both NHS and private facilities for the diagnosis. 23% used exclusively public facilities, while 22% used exclusively private facilities. (**Figure 1C**). With regard to the course of treatment, the sample appears to be more evenly distributed among those who used both public and private facilities (39%), those who relied exclusively on public care (30%), and those who used only private providers (31%). (**Figure 1D**).

**Figure 3:**
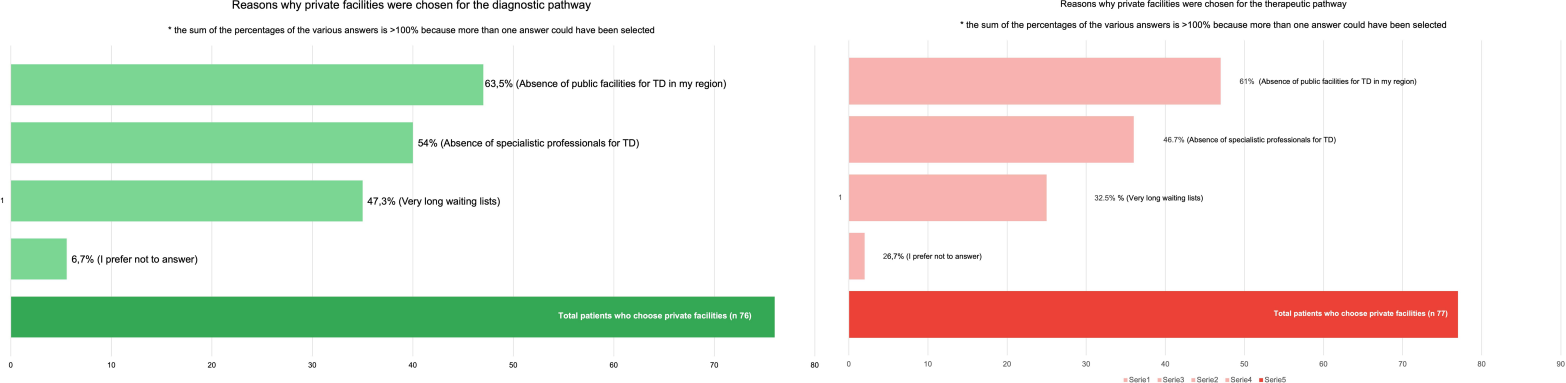
In this figure are showed all the reasons why our patients choose private facilities instead of public services.

The reasons why private facilities were chosen were also investigated, with most of the sample (62% for both diagnosis and therapy) reporting that public services for TS/TD were not available in their region. Other reasons are reported in **Figures 3A, B**. **Table 2** presents data on how participants perceive the availability and clarity of information regarding the diagnostic and therapeutic process for TS/TD. The lack of clarity in available information is a major concern, despite caregivers reporting easier access to information than youths and adult participants with TS/TD (p= .042, OR: 0.37). The quality of the diagnostic and therapeutic process was perceived as moderate, with no significant difference emerging based on whether it was performed in a private or public setting (**Table 2**).

**Table 2:** Responses about the availability of information regarding the therapeutic-diagnostic process for Tourette syndrome and other tic disorders.

Comments	Total sample (n = 100)	Youth and Adults *^1^* (n = 74)	Children and Early Adolescence *^2^* (n = 26)
*Comments about the availability of information*			
Easy to find *- %*	30	22.9*	42.3*
Difficult to find *- %*	25	28.4	11.5
Clear to understand *- %*	0	0	0
Not clear to understand *- %*	14	12.2	15.3
Poorly available *- %*	41	41.8	11.5
*Comments about quality*			
Diagnostic process (0-5) *- mean (SD)*	3.40 (1.08)	3.32 (1.08)	3.62 (1.06)
Therapeutic process (0-5) *- mean (SD)*	3.59 (1.11)	3.62 (1.09)	3.50 (1.18)
Transition from pediatric to adult care services (0-5) *- mean (SD)*	/	3.06 (1.44)	/

^1^ Patients older than 14 years of age; ^2^ Patients younger than 14 years of age.

* Statistically significant differences after chi-squared tests between the two subgroups.

Additionally, adult patients were asked to rate the perceived difficulty of the transition from pediatric to adult care services. This transition was perceived as difficult by the majority of them (68.7%).

### 3.3 Lifetime therapeutic interventions

In our sample, pharmacological therapy was the most commonly received treatment, with over half of the total sample currently under treatment (53%), and an even higher percentage having received it in the past (63%). Currently, psychotherapy is more commonly received by children and early adolescents (50%) compared to youth and adults (25.6%) (p = .037). However, a similar percentage of youth/adults (50%) and children/adolescents (53.8%) had undergone psychotherapy at some point in their lives. Habit-reversal training (HRT) was relatively uncommon, with only 7% of the total sample currently receiving it. A higher percentage of children and early adolescents (24%) had received HRT in the past compared to youth and adults (14.8%). No participant reported ever having been subjected to Exposure-Prevention Therapy (EPT), either currently or in the past (**Table 3**).

**Table 3:** Type of therapeutic interventions in the sample.

Timing	Total sample (n = 100)	Youth and Adults*¹* (n = 74)	Children and Early Adolescence*²* (n = 26)
*Psychotherapy*			
current - *%*	32.6	25.6*	50*
lifetime - *%*	50.5	50	53.8
- *Habit-reversal training*			
current - *%*	7	6,7	7.7
lifetime - *%*	18	14.8	24
- *Exposure-prevention therapy*			
current - *%*	0	0	0
lifetime - *%*	0	0	0
*Pharmacological therapy*			
current - *%*	53	52.7	61.5
lifetime *%*	63	60.8	65.3

¹ Patients older than 14 years of age; ² Patients younger than 14 years of age.

* Statistically significant differences after independent t-test between the two subgroups.

### 3.4 Impact on functioning and quality of life

The principal features of tics are described in **Table 4**, showing no significant differences among the age groups. Tic symptoms significantly impacted the QoL across social, school, and work functioning, with similar effects across all ages, while sentimental life was less affected. The impact of comorbidities on QoL - despite being equally distributed between the age groups - was significantly more relevant than impact of tics on functioning (p= .004, Cohen’s d=0.3).

**Table 4:** Characteristics of tics and impact on Quality of Life (QoL).

	Total sample (n = 100)	Youth and Adults*¹* (n = 74)	Children and Early Adolescence*²* (n = 26)
*Motor Tics*			
Multiple tics (two or more) - *%*	84	78.3	84.6
Complexity (0-3) *- mean (SD)*	1.42 (1.04)	1.38 (0.96)	1.54 (1.25)
Disability (0-4) *- mean (SD)*	1.43 (1.20)	1.39 (1.20)	1.54 (1.25)
*Phonic Tics*			
Multiple tics (two or more) - *%*	53	48.7	53.8
Complexity (0-3) *- mean (SD)*	1.17 (1.05)	1.12 (0.96)	1.33 (1.28)
Disability (0-4) *- mean (SD)*	1.18 (1.23)	1.12 (1.16)	1.38 (1.47)
*Impact of tics lifelong*			
Social functioning (0-5) *- mean (SD)*	3.34 (1.47)	3.32 (1.47)	3.38 (1.50)
School functioning (0-5) *- mean (SD)*	3.14 (1.70)	3.00 (1.72)	3.54 (1.64)
Work functioning (adults) (0-5) *- mean (SD)*	2.41 (1.72)	2.41 (1.72)	–
Sentimental life (0-3) *- mean (SD)*	1.66 (1.06)	1.66 (1.06)	–
Overall impact on QoL (0-5) *-mean (SD)*	3.17 (1.41)	3.07 (1.40)	3.46 (1.44)
*Comorbidities*			
Number of comorbidities *- mean (SD)*	1.44 (1.03)	1.44 (1.10)	1.46 (0.78)
Impact on functioning *lifelong* (0-5) *- mean (SD)*	3.57 (1.61)	3.45 (1.65)	3.92 (1.47)
*Stigmatization*			
Absence of stigmatization - *%*	67	66.2	69.2
Some degrees of stigmatization - *%*	28	29.7	23.1

¹ Patients older than 14 years of age; ² Patients younger than 14 years of age.

Furthermore, no significant correlations were found between stigma and other variables, such as age, comorbidities, overall impact of tics, impact of comorbidities, and tic severity. Notably, 67% of our sample did not refer to being a victim of social stigmatization.

## 4 Discussion

Access to timely and specialized care is a critical factor influencing the diagnostic and therapeutic journey of individuals with TS/TD. Despite the availability of public healthcare, many families seek private services, often due to limited specialized resources and long waiting times. These barriers contribute to diagnostic delays and deviations from recommended treatment guidelines, ultimately impacting patients’ QoL and long-term outcomes.

### 4.1 Diagnostic process and accessibility to care

Access to care — defined as the ease of accessing health services in terms of geographical distribution, cost, time, and workforce availability (24) — is a crucial factor affecting TS/TD patients and their families, starting from the very first step of their journey: obtaining a diagnosis.

Despite the NHS offering free or low-cost public healthcare, only 23% of our sample relied exclusively on NHS facilities for diagnosis. Instead, the majority of families sought care from private providers or a combination of public and private services. This preference for private healthcare is particularly striking given the financial constraints reported by many families, with nearly 58% experiencing some degree of economic hardship in accessing care. These findings align with data from the European Patients’ Forum (15), which indicates that 60% of patients encounter financial barriers to healthcare, particularly those with multiple comorbidities (15).

Interestingly, despite the widespread use of private healthcare, there were no significant differences in the perceived quality of these services compared to public facilities. Our findings suggest that the primary motivation for choosing private providers is the lack of adequate public facilities for this specific condition. Existing literature further highlights persistent challenges in securing timely and affordable care for individuals with TS/TD, likely due to the limited availability of specialized services and trained professionals within the public healthcare sector (17, 25).

These challenges are further exacerbated by the lack of clear and accessible information regarding diagnostic procedures. Indeed, this was a significant concern for most respondents to our survey, with no participants finding the information entirely clear or easy to understand. However, caregivers reported fewer difficulties accessing information for their children than adult patients. This discrepancy may be attributed to generational differences, as information about TS/TD has likely become more widely available in recent years, facilitating access to appropriate care for parents of children with TS/TD (25). Previous studies (11, 24) investigated these topics and reported that the most frequent concerns were lack of transparency regarding costs, poor accessibility for people with disabilities, and difficulties in understanding diagnostic/therapeutic procedures (11, 25). Additionally, an analysis of widely used patient information leaflets on TS/TD identified several limitations, including the absence of explicit recommendations to consult a neuropsychiatrist for diagnostic evaluation (26).

The challenges associated with accessing the diagnostic process for TS/TD have significant negative consequences, primarily contributing to prolonged delays between symptom onset, initial clinical consultation, and final diagnosis. In our sample, the mean time from symptom onset to a formal TS/TD diagnosis was 7.7 years, consistent with previous studies reporting diagnostic delays ranging from 3 to 8 years (25). This delay could be explained also by the initial misdiagnosis with other neurodevelopmental disorders, behavioral problems (27) or pediatric conditions (e.g. asthma or allergies) (28). Notably, this delay was more pronounced in older patients, with an average of 9.3 years compared to 3.5 years in younger patients which is consistent with available literature (29).

### 4.2 Lifetime therapeutic interventions

Clinical guidelines for the treatment of TS/TD advocate a stepwise approach, with psychoeducation and behavioral interventions as first-line strategies and pharmacotherapy reserved for third-line use (16). However, in our sample, only about 50% of individuals have undergone psychotherapy—and notably, none received specific intervention for TS/TD such as EPT. Among those who never received any psychotherapeutic intervention, 13.4% were treated exclusively with medication. This deviation from the guidelines suggests that treatment choices are probably driven by the immediate availability and perceived convenience of pharmacological options, rather than the structured, time-intensive nature of behavioral therapies (30, 31). Moreover, barriers such as limited access to well-trained practitioners, high costs of psychotherapy, and insufficient patient education further contribute to this practice (26, 32).

### 4.3 Quality of life: the impact of tics and comorbidities

The impact of tics on global QoL—including social, academic, occupational, and sentimental functioning—was assessed, showing significant impairment in patients with TS/TD. All domains were similarly affected, with comparable distribution across age groups. Previous studies suggested that children with TS/TD experience higher rates of social difficulties, such as bullying, peer rejection, and challenges in forming and maintaining friendships compared to neurotypical peers (6). In this context, it is important to consider the role of comorbidities, which often exacerbate social difficulties in children with TS/TD (33). In our cohort, the most common comorbidity was ADHD, followed by mood disorders. Anxiety and depression were the most frequent comorbid symptoms, followed by inattention and impulsivity. Only five patients reported no additional symptoms, underlining the high prevalence of concurrent conditions in TS/TD (7, 34). The impact of comorbidities on overall functioning was perceived as high, with their effect on QoL being more pronounced than the global impact of tics, as shown in previous studies (35, 36), which underscores the substantial burden of comorbidities in TS/TD (37).

Another important issue is that TS/TD is frequently misunderstood, leading to social stigma and discrimination. This phenomenon is not only prevalent in social contexts but also among healthcare professionals, who may hold unhelpful beliefs about the condition (38). In our survey, nearly 30% experienced inappropriate comments or behaviors; however these occurrences might have been underestimated in caregiver reports.

Finally, the transition from child and adolescent neuropsychiatry to adult services also was perceived as a significant challenge within our population. The majority of individuals reported having faced some or great difficulties in continuing their care after reaching adulthood, due to complex bureaucracy. Previous studies suggested that the transition from child to adult healthcare services is often poorly planned and executed, leading to challenges such as lack of continuity, inadequate support, and increased anxiety, highlighting the need for improved transition models and psychological interventions (39).

## 5 Limitation and future directions

Although all participants reported receiving a diagnosis of TS or TD from a specialist, the self-reported nature of our survey prevents us from verifying the specific diagnostic criteria used in each case.

Patients were not selected based on their primary diagnosis because comorbidities, which can significantly impact functional outcomes, may be underreported in self-report surveys. Excluding individuals with some kind of comorbidities could have overlooked those with TS/TD who experience a greater negative impact on their QoL due to these conditions.

In our sample, the child and adolescent population was less represented, limiting the possibility of generalizing the results obtained in this age group. Further studies on a larger sample might make it possible to overcome this limitation.

For future research, it would be beneficial to adopt a more precise patient stratification, potentially by focusing solely on individuals diagnosed by clinicians. This would also allow psychiatric and internal medicine comorbidities to be investigated more accurately, as well as the different types of drug therapy administered. However, this approach could limit the survey’s reach and unintentionally disadvantage patients who face greater challenges in accessing specialist care—a group that was notably represented in our study.

## 6 Conclusion

Our study underscores significant barriers to accessing specialized care for individuals with TS/TD, despite the availability of public healthcare in our country. Limited specialized services, financial constraints, and unclear diagnostic information contribute to prolonged diagnostic delays—particularly among older patients—and drive many families to seek private care.

Notably, deviations from clinical guidelines are relevant: only about half of the patients received psychotherapy during their lifetime, while a notable proportion relied exclusively on pharmacotherapy. This pattern suggests that treatment choices are more influenced by the immediate availability of medication than by evidence-based, stepwise interventions. Of particular significance, the use of specialized cognitive-behavioral interventions for TS/TD — specifically, EPT and HRT — was exceedingly rare, with only 7% of patients receiving HRT and none undergoing EPT, regardless of geographic location.

Finally, the substantial impact of TS/TD on QoL, compounded by frequent comorbidities and challenges in transitioning from pediatric to adult services, highlights the urgent need for improved care models, enhanced provider training, and better patient education to facilitate timely diagnosis and effective, integrated treatment strategies.

## Data Availability

The raw data supporting the conclusions of this article will be made available by the authors, without undue reservation.
