# Supplementary figures and images for: Growing up with tic disorders: an Italian survey on quality of life and access to care
Source: Front Psychiatry. 2025 May 13;16:1581666. doi: 10.3389/fpsyt.2025.1581666 (PMC12106353; doi:10.3389/fpsyt.2025.1581666)

# Comorbid symptoms

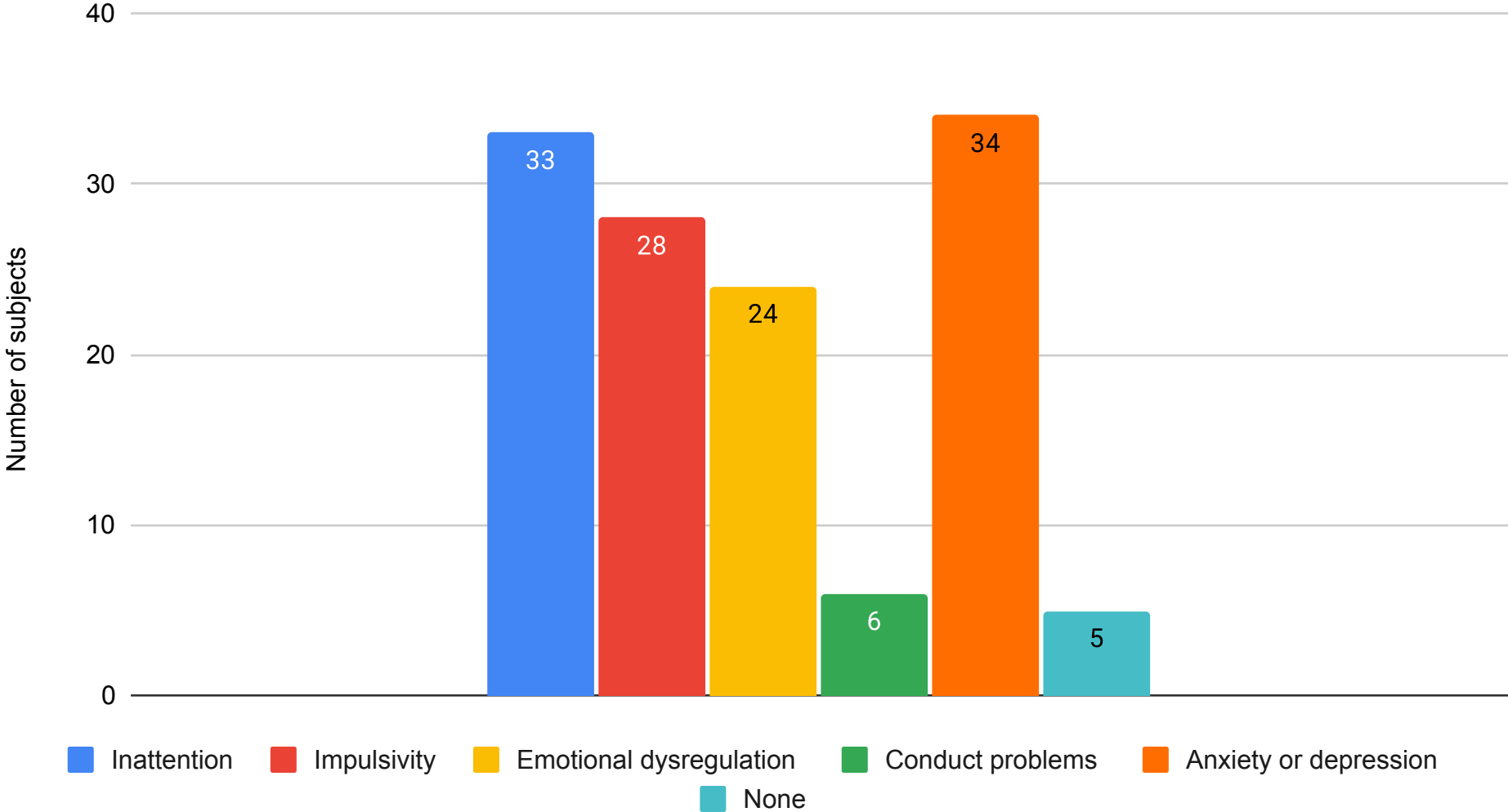

Supplement: Supplementary file 1 [file DataSheet1.pdf]
