# Supplementary material for: Growing up with tic disorders: an Italian survey on quality of life and access to care
Source: Front Psychiatry. 2025 May 13;16:1581666. doi: 10.3389/fpsyt.2025.1581666 (PMC12106353; doi:10.3389/fpsyt.2025.1581666)
Supplement: Supplementary file 2 [file Table1.docx]

***Supplementary Material***

# Survey administered in our cohort

### Tourette Syndrome - Impairment and Quality of Life

**General Information**

Dear Sir/Madam,

This questionnaire is part of a clinical research project conducted by the "Giovanni Bollea" Institute of Child Neuropsychiatry (Policlinico Umberto I, Sapienza University of Rome).
It concerns the impact of Tourette Syndrome on daily life, both in childhood and adulthood and is addressed to adults with Tourette Syndrome and parents/legal guardians of children with Tourette Syndrome (aged over 13 years).

The questionnaire includes a brief section with general information about yourself or your child, followed by questions about your or your child's symptoms, diagnostic and therapeutic journey, and finally, questions about quality of life and certain habits.

The aim of this questionnaire is to help us better understand the impact that Tourette Syndrome can have on the quality of life and psychological development of individuals with the condition. Completing it will take approximately 15 minutes.

All information collected in this research will be treated in compliance with the applicable laws (Legislative Decree 196/2003 on privacy and GDPR 679/2016 on personal data protection) and the ethical codes of the Italian Orders of Doctors and Psychologists. The data will be used exclusively for the purposes of this research.

The research is overseen by Dr. Valentina Baglioni of the Child Neuropsychiatry Unit at Policlinico Umberto I, Sapienza University of Rome. Dr. Baglioni commits to fulfilling all obligations related to the collection, processing, and storage of sensitive data in accordance with current regulations.

Each participant retains the right to exercise their rights under Article 7 of Legislative Decree 196/2003 at any time. The collected data may be included in publications and/or presented at scientific congresses or seminars in an aggregated and anonymous form.

Data processing will commence only upon the signing of consent.

For further clarification, you may contact Dr. Giorgia Di Iorio, Dr. Sara Romano, or Dr. Dario Esposito at the following addresses:

- [giorgia.diiorio@uniroma1.it](mailto:giorgia.diiorio@uniroma1.it)
- [ilaria.notaristefano@uniroma1.it](mailto:ilaria.notaristefano@uniroma1.it)
- [sara.romano@uniroma1.it](mailto:sara.romano@uniroma1.it)
- [dario.esposito@uniroma1.it](mailto:dario.esposito@uniroma1.it)

Thank you for participating.

**1. Questionnaire Section: Who is responding?**

- I am an adult with Tourette Syndrome.
- I am the parent/legal guardian of a minor with Tourette Syndrome. *(If selected, proceed to question 41.)*

**2. What is your gender?**

- Male
- Female
- Other (please specify):

**3. Which gender do you identify with?**

- Male
- Female
- Prefer not to answer

**4. Date of birth***(Example: January 7, 2019)*

**5. What is your region of residence?***(Select one option)*

- Piedmont
- Valle d'Aosta
- Lombardy
- Trentino-Alto Adige
- Veneto
- Friuli-Venezia Giulia
- Liguria
- Emilia-Romagna
- Tuscany
- Umbria
- Marche
- Lazio
- Abruzzo
- Molise
- Campania
- Apulia
- Basilicata
- Calabria
- Sicily
- Sardinia

**6. Do you live in an urban or rural area?**

- Urban (city, suburb, medium-large town)
- Rural (open countryside, village/small town)

**7. At what age did you start showing the first symptoms of Tourette Syndrome?***(Provide the age in years.)*

### Symptomatology Overview

**8. Symptomatology Detail**Respond to the following questions referring to your symptoms.

**9. Number of Tics***(Select one option for each row)*

| **Tic Type** | **None** | **Single Tic** | **Multiple Tics (2-5)** |
| --- | --- | --- | --- |
| Motor | ☐ | ☐ | ☐ |
| Vocal | ☐ | ☐ | ☐ |

**10. Complexity***(Select one option for each row)*

| **Tic Type** | **None** | **Mild** | **Moderate** | **Severe** |
| --- | --- | --- | --- | --- |
| Motor | ☐ | ☐ | ☐ | ☐ |
| Vocal | ☐ | ☐ | ☐ | ☐ |

- **None:** If present, all tics are "simple" (sudden, brief, and purposeless).
- **Mild:** Some tics are clearly "complex" (apparently purposeful) and mimic automatic behaviors like cleaning oneself or uttering syllables/short phrases that can be easily camouflaged.
- **Moderate:** Some tics are more "complex" (more purposeful and prolonged) and may occur as difficult-to-camouflage episodes.
- **Severe:** Some tics involve long episodes of organized behaviors or phrases that are impossible to camouflage or rationalize as normal.

**11. Interference***(Select one option for each row)*

| **Tic Type** | **None** | **Mild** | **Moderate** | **Severe** |
| --- | --- | --- | --- | --- |
| Motor | ☐ | ☐ | ☐ | ☐ |
| Vocal | ☐ | ☐ | ☐ | ☐ |

- **None:** When tics are present, they do not interrupt normal behavior or speech.
- **Mild:** When tics are present, they occasionally interrupt normal behavior or speech.
- **Moderate:** When tics are present, they frequently interrupt normal behavior or speech.
- **Severe:** When tics are present, they frequently interrupt voluntary actions or communication.

**12. Disability***(Select one option for each row)*

| **Tic Type** | **None** | **Mild** | **Moderate** | **Severe** |
| --- | --- | --- | --- | --- |
| Motor | ☐ | ☐ | ☐ | ☐ |
| Vocal | ☐ | ☐ | ☐ | ☐ |

- **None:** Tics are not associated with difficulties in self-esteem, family life, social acceptance, or school/work functioning.
- **Mild:** Tics are associated with slight difficulties in self-esteem, family life, social acceptance, or school/work functioning.
- **Moderate:** Tics are associated with significant difficulties in self-esteem, family life, social acceptance, or school/work functioning (e.g., episodes of dysphoria, periodic family crises, frequent teasing by peers, occasional social avoidance, interference with academic/work performance).
- **Severe:** Tics are associated with marked difficulties in self-esteem, family life, social acceptance, or school/work functioning (e.g., severe depression, suicidal ideation, family separation/divorce, residential changes, or severe social restrictions).

### Social and Academic Impact

**13. To what extent do you feel that these tics have impacted your social life?** *(Rate from 0 to 5)*

- Not at all: 0
- Very much: 5

**14. Select all applicable options regarding your social life:**

- It has been very difficult to make friends because of Tourette Syndrome.
- I have felt lonely because of Tourette Syndrome.
- I have been teased because of Tourette Syndrome.
- Tourette Syndrome has not had any impact on my social life.

**15. To what extent do you feel that these tics have impacted your academic/university life?** *(Rate from 0 to 5)*

- Not at all: 0
- Very much: 5

**16. Select all applicable options regarding your academic/university life:**

- It has been very difficult to attend school because of Tourette Syndrome.
- I have dropped out of school because of Tourette Syndrome.
- I have not been allowed to attend classes/labs/internships because of Tourette Syndrome.
- I have faced difficulties enforcing specific accommodations for my condition regarding learning methods (e.g., a personalized educational plan).
- Tourette Syndrome has not had any impact on my academic/university life.

**17. To what extent do you feel that these tics have impacted your work life?** *(Rate from 0 to 5)*

- Not at all: 0
- Very much: 5

**18. Select all applicable options regarding your work life:**

- It has been very difficult to find a job because of Tourette Syndrome.
- I have lost many jobs/opportunities because of Tourette Syndrome.
- Tourette Syndrome has not had any impact on my work life.
- I have not yet had work experience.

### Romantic and Emotional Life

**19. How satisfied are you with your romantic/emotional life?** *(Rate from 1 to 5)*

- Very low satisfaction: 1
- Very high satisfaction: 5

**20. Have you/Do you experience difficulties finding a partner?**

- Yes
- No
- Prefer not to answer

**21. Do you think that your Tourette condition has influenced your ability to find or maintain a romantic relationship?**

- Yes, it has influenced me in the past and still does.
- Yes, it has influenced me in the past only.
- No, it has never influenced me.
- Prefer not to answer.

**Comorbidities and Current Treatment**

**22. In association with tic symptoms, do you experience any of the following?** *(Select all applicable options)*

- Easy distractibility
- Impulsiveness
- Anger outbursts
- Conduct problems (e.g., suspensions or notes from school, exclusion from sports activities, fights)
- Anxiety or mood problems (e.g., panic attacks, depression, social withdrawal)
- None of the above

**23. Beyond your Tourette Syndrome diagnosis, do you have any of the following neuropsychiatric conditions?** *(Select all applicable options)*

- None
- ADHD (Attention Deficit Hyperactivity Disorder)
- Specific Learning Disorder
- Developmental Coordination Disorder
- Autism Spectrum Disorder
- Disruptive Mood Dysregulation Disorder
- Anxiety or Mood Disorder
- OCD (Obsessive-Compulsive Disorder)

**24. To what extent do you feel that these comorbidities have impacted your social, academic, or work life?** *(Rate from 0 to 5)*

- Not at all: 0
- Very much: 5

**25. Are you currently undergoing treatment for Tourette Syndrome?** *(Select all applicable options)*

- No, I am not undergoing any treatment.
- Individual psychotherapy.
- Habit reversal training.
- Exposure prevention therapy.
- Pharmacological therapy (e.g., aripiprazole, risperidone, antidepressants).

**26. Have you undergone treatment for Tourette Syndrome in the past?** *(Select all applicable options)*

- No, I have never undergone any treatment.
- Individual psychotherapy.
- Habit reversal training.
- Exposure prevention therapy.
- Pharmacological therapy (e.g., aripiprazole, risperidone, antidepressants).

### Experience with the National Healthcare System (NHS) - Diagnostic Journey

**27. How easy was it to access the necessary medical consultations for a diagnosis of Tourette Syndrome?**

- Very easy
- Easy
- With some difficulty
- With many difficulties
- I don’t know
- Prefer not to answer

**28. How did you undergo the necessary medical consultations for a diagnosis of Tourette Syndrome?**

- Public sector (territorial services, hospitals, public outpatient clinics)
- Private sector (private medical consultations, private clinics)
- Both public and private sector

**29. If you underwent consultations in the private sector, what was the reason for this choice?***(Select all applicable options)*

- Due to excessively long waiting times in public facilities
- Due to the absence of adequate public facilities for my condition
- Due to the lack of suitable specialists for my condition
- I did not undergo private-sector consultations
- Prefer not to answer

**30. How would you describe the availability of information about the correct diagnostic process for Tourette Syndrome?***(e.g., which public facilities to approach for medical consultations, how to access waiting lists, how to communicate with healthcare facilities)*

- Readily available and easy to find (e.g., websites, helpline numbers, posters, etc.)
- Available but difficult to find/understand
- Scarcely available
- Clear in terms of costs/timelines
- Unclear in terms of costs/timelines

**31. Would you say that the healthcare facilities you visited for your diagnostic journey are sufficiently close to your home?**

- Yes, I can reach them comfortably.
- No, reaching them requires a lot of time.
- No, I have to travel to another province or region.
- No, I had to relocate due to the distance.

**32. Have you ever felt stigmatized or discriminated against during your diagnostic journey? If yes, what kind of experience did you have?***(Stigma refers to the set of negative prejudices attributed to individuals because of their condition, resulting in rejection, discrimination, and exclusion.)
(Select all applicable options)*

- No, I have not experienced stigma or discrimination.
- Yes, inappropriate behavior from healthcare staff.
- Yes, denial of my rights.
- Yes, inappropriate language or comments.
- Yes, lack of healthcare facilities in my community.
- Yes, refusal to provide me with care.

**33. How would you rate the quality of care you received during your diagnostic journey?***(Rate from 1 to 5)*

- Very poor: 1
- Excellent: 5

### Experience with the National Healthcare System (NHS) - Therapeutic Journey

**34. How would you describe the transition from pediatric to adult care in the public sector?***(Refer to the bureaucratic procedures required upon turning 18 to transition from child to adult territorial services.)
(Rate from 1 to 5)*

- Very simple: 1
- Very complex: 5

**35. How did you undergo your therapeutic journey for Tourette Syndrome?**

- Public sector (territorial services, hospitals, public outpatient clinics)
- Private sector (private medical consultations, private clinics)
- Both public and private sector

**36. If you underwent your therapeutic journey in the private sector, what was the reason for this choice?***(Select all applicable options)*

- Due to excessively long waiting times in public facilities
- Due to the absence of adequate public facilities for my condition
- Due to the lack of suitable specialists for my condition
- I did not undergo a therapeutic journey in the private sector
- Prefer not to answer

**37. Would you say that the healthcare facilities you visited for your therapeutic journey are sufficiently close to your home?**

- Yes, I can reach them comfortably.
- No, reaching them requires a lot of time.
- No, I have to travel to another province or region.
- No, I had to relocate due to the distance.

**38. How would you rate the quality of care you received during your therapeutic journey?***(Rate from 1 to 5)*

- Very poor: 1
- Excellent: 5

**39. How satisfied are you with the quality and safety of the care you received?***(Select one option for each row)*

| **Statement** | **Always** | **Often** | **Rarely** | **Never** | **Don’t know** |
| --- | --- | --- | --- | --- | --- |
| I receive care of good quality according to the standards/guidelines or best practices available for my condition. | ☐ | ☐ | ☐ | ☐ | ☐ |
| I am satisfied with the safety of the care I received. | ☐ | ☐ | ☐ | ☐ | ☐ |
| I am satisfied with the continuity of care over time. | ☐ | ☐ | ☐ | ☐ | ☐ |

**40. Are you able to afford the costs associated with your therapy?**

- Yes, without difficulty.
- Yes, thanks to state subsidies/reimbursements.
- No, I face significant difficulties.
- Prefer not to answer.

###

###

### For Parents/Legal Guardians of Minors with Tourette Syndrome

**1. What is the gender of your child?**

- Male
- Female

**2. Which gender does your child identify with?**

- Male
- Female
- Prefer not to answer
- Other (please specify):

**3. Date of birth of your child** *(Example: January 7, 2019)*

**4. What is your region of residence?** *(Select one option)*

- Piedmont
- Valle d'Aosta
- Lombardy
- Trentino-Alto Adige
- Veneto
- Friuli-Venezia Giulia
- Liguria
- Emilia-Romagna
- Tuscany
- Umbria
- Marche
- Lazio
- Abruzzo
- Molise
- Campania
- Apulia
- Basilicata
- Calabria
- Sicily
- Sardinia

**5. Do you live in an urban or rural area?**

- Urban (city, suburb, medium-large town)
- Rural (open countryside, village/small town)

**6. At what age did your child start showing the first symptoms of Tourette Syndrome?** *(Provide the age in years.)*

### Symptomatology Overview

**7. Symptomatology Detail** Answer the following questions referring to your child's symptoms.

**8. Number of Tics** *(Select one option for each row)*

| **Tic Type** | **None** | **Single Tic** | **Multiple Tics (2-5)** |
| --- | --- | --- | --- |
| Motor | ☐ | ☐ | ☐ |
| Vocal | ☐ | ☐ | ☐ |

**9. Complexity** *(Select one option for each row)*

| **Tic Type** | **None** | **Mild** | **Moderate** | **Severe** |
| --- | --- | --- | --- | --- |
| Motor | ☐ | ☐ | ☐ | ☐ |
| Vocal | ☐ | ☐ | ☐ | ☐ |

Definitions are the same as for adults:

- **None, Mild, Moderate, Severe:** Detailed in earlier sections, with specific examples of tic complexity and their effect on daily life.

**10. Interference** *(Select one option for each row)*

| **Tic Type** | **None** | **Mild** | **Moderate** | **Severe** |
| --- | --- | --- | --- | --- |
| Motor | ☐ | ☐ | ☐ | ☐ |
| Vocal | ☐ | ☐ | ☐ | ☐ |

- **None:** Tics do not interrupt behavior or speech.
- **Mild:** Tics occasionally interrupt normal behavior or speech.
- **Moderate:** Tics frequently interrupt behavior or speech.
- **Severe:** Tics frequently disrupt voluntary actions or communication.

**11. Disability** *(Select one option for each row)*

| **Tic Type** | **None** | **Mild** | **Moderate** | **Severe** |
| --- | --- | --- | --- | --- |
| Motor | ☐ | ☐ | ☐ | ☐ |
| Vocal | ☐ | ☐ | ☐ | ☐ |

Definitions follow the same framework as earlier:

- **None, Mild, Moderate, Severe:** Levels of impact on self-esteem, family life, social acceptance, or school functioning. Severe levels may include depression, suicidal ideation, or significant social restrictions.

### Social and Academic Impact for Minors

**12. To what extent do you feel that your child’s tics have impacted their social life?** *(Rate from 0 to 5)*

- Not at all: 0
- Very much: 5

**13. Select all applicable options regarding your child’s social life:**

- It has been very difficult for my child to make friends because of Tourette Syndrome.
- My child has felt lonely because of Tourette Syndrome.
- My child has been teased because of Tourette Syndrome.
- Tourette Syndrome has not had any impact on my child’s social life.

**14. To what extent do you feel that your child’s tics have impacted their academic/school life?** *(Rate from 0 to 5)*

- Not at all: 0
- Very much: 5

**15. Select all applicable options regarding your child’s academic/school life:**

- It has been very difficult for my child to attend school because of Tourette Syndrome.
- My child dropped out of school because of Tourette Syndrome.
- My child was not allowed to attend classes/labs/internships because of Tourette Syndrome.
- I have had difficulty enforcing specific accommodations for my child’s condition regarding learning methods (e.g., personalized educational plan).
- Tourette Syndrome has not had any impact on my child’s academic life.

### Comorbidities and Current Treatment for Minors

**16. In association with tic symptoms, does your child experience any of the following?** *(Select all applicable options)*

- Easy distractibility
- Impulsiveness
- Anger outbursts
- Conduct problems (e.g., suspensions or notes from school, exclusion from sports activities, fights)
- Anxiety or mood problems (e.g., panic attacks, depression, social withdrawal)
- None of the above

**17. Beyond the Tourette Syndrome diagnosis, does your child have any of the following neuropsychiatric conditions?** *(Select all applicable options)*

- None
- ADHD (Attention Deficit Hyperactivity Disorder)
- Specific Learning Disorder
- Developmental Coordination Disorder
- Autism Spectrum Disorder
- Disruptive Mood Dysregulation Disorder
- Anxiety or Mood Disorder
- OCD (Obsessive-Compulsive Disorder)

**18. To what extent do you feel that these comorbidities have impacted your child’s social or academic life?** *(Rate from 0 to 5)*

- Not at all: 0
- Very much: 5

**19. Is your child currently undergoing treatment for Tourette Syndrome?** *(Select all applicable options)*

- No, they are not undergoing any treatment.
- Individual psychotherapy.
- Habit reversal training.
- Exposure prevention therapy.
- Pharmacological therapy (e.g., aripiprazole, risperidone, antidepressants).

**20. Has your child undergone treatment for Tourette Syndrome in the past?** *(Select all applicable options)*

- No, they have never undergone any treatment.
- Individual psychotherapy.
- Habit reversal training.
- Exposure prevention therapy.
- Pharmacological therapy (e.g., aripiprazole, risperidone, antidepressants).

### Experience with the National Healthcare System (NHS) - Diagnostic Journey

**21. How easy was it to access the necessary medical consultations for your child’s diagnosis of Tourette Syndrome?**

- Very easy
- Easy
- With some difficulty
- With many difficulties
- I don’t know
- Prefer not to answer

**22. How did your child undergo the necessary medical consultations for a diagnosis of Tourette Syndrome?**

- Public sector (territorial services, hospitals, public outpatient clinics)
- Private sector (private medical consultations, private clinics)
- Both public and private sector

**23. If your child underwent consultations in the private sector, what was the reason for this choice?** *(Select all applicable options)*

- Due to excessively long waiting times in public facilities
- Due to the absence of adequate public facilities for their condition
- Due to the lack of suitable specialists for their condition
- My child did not undergo private-sector consultations
- Prefer not to answer

**24. How would you describe the availability of information about the correct diagnostic process for Tourette Syndrome?** *(e.g., which public facilities to approach for medical consultations, how to access waiting lists, how to communicate with healthcare facilities)*

- Readily available and easy to find (e.g., websites, helpline numbers, posters, etc.)
- Available but difficult to find/understand
- Scarcely available
- Clear in terms of costs/timelines
- Unclear in terms of costs/timelines

**25. Would you say that the healthcare facilities you visited for your child’s diagnostic journey are sufficiently close to your home?**

- Yes, I can reach them comfortably.
- No, reaching them requires a lot of time.
- No, I have to travel to another province or region.
- No, I had to relocate due to the distance.

**26. Has your child ever felt stigmatized or discriminated against during their diagnostic journey? If yes, what kind of experience did they have?** *(Stigma refers to the set of negative prejudices attributed to individuals because of their condition, resulting in rejection, discrimination, and exclusion.)* *(Select all applicable options)*

- No, my child has not experienced stigma or discrimination.
- Yes, inappropriate behavior from healthcare staff.
- Yes, denial of their rights.
- Yes, inappropriate language or comments.
- Yes, lack of healthcare facilities in our community.
- Yes, refusal to provide them with care.

**27. How would you rate the quality of care your child received during their diagnostic journey?** *(Rate from 1 to 5)*

- Very poor: 1
- Excellent: 5

### Experience with the National Healthcare System (NHS) - Therapeutic Journey

**28. How did your child undergo their therapeutic journey for Tourette Syndrome?**

- Public sector (territorial services, hospitals, public outpatient clinics)
- Private sector (private medical consultations, private clinics)
- Both public and private sector

**29. If your child underwent their therapeutic journey in the private sector, what was the reason for this choice?** *(Select all applicable options)*

- Due to excessively long waiting times in public facilities
- Due to the absence of adequate public facilities for their condition
- Due to the lack of suitable specialists for their condition
- My child did not undergo a therapeutic journey in the private sector
- Prefer not to answer

**30. Would you say that the healthcare facilities you visited for your child’s therapeutic journey are sufficiently close to your home?**

- Yes, I can reach them comfortably.
- No, reaching them requires a lot of time.
- No, I have to travel to another province or region.
- No, I had to relocate due to the distance.

**31. How would you rate the quality of care your child received during their therapeutic journey?** *(Rate from 1 to 5)*

- Very poor: 1
- Excellent: 5

**32. Are you satisfied with the quality and safety of your child’s care?** *(Select one option for each row)*

| **Statement** | **Always** | **Often** | **Rarely** | **Never** | **Don’t know** |
| --- | --- | --- | --- | --- | --- |
| My child receives care of good quality according to the standards/guidelines or best practices available for their condition. | ☐ | ☐ | ☐ | ☐ | ☐ |
| I am satisfied with the safety of the care my child received. | ☐ | ☐ | ☐ | ☐ | ☐ |
| I am satisfied with the continuity of care over time. | ☐ | ☐ | ☐ | ☐ | ☐ |

**33. Are you able to afford the costs associated with your child’s therapy?**

- Yes, without difficulty.
- Yes, thanks to state subsidies/reimbursements.
- No, I face significant difficulties.
- Prefer not to answer.
